# Effectiveness of a Pre-treatment Snack on the Uptake of Mass Treatment for Schistosomiasis in Uganda: A Cluster Randomized Trial

**DOI:** 10.1371/journal.pmed.1001640

**Published:** 2014-05-13

**Authors:** Simon Muhumuza, Annette Olsen, Anne Katahoire, Agnes N. Kiragga, Fred Nuwaha

**Affiliations:** 1 Makerere University, School of Medicine, Child Health and Development Center, Kampala, Uganda; 2 University of Copenhagen, Faculty of Health and Medical Sciences, Section for Parasitology, Health and Development, Copenhagen, Denmark; 3 Infectious Diseases Institute, College of Health Sciences, Makerere University, Kampala, Uganda; 4 Makerere University, School of Public Health, Kampala, Uganda; St. George's, University of London, United Kingdom

## Abstract

**Background:**

School-based mass treatment with praziquantel is the cornerstone for schistosomiasis control in school-aged children. However, uptake of treatment among school-age children in Uganda is low in some areas. The objective of the study was to examine the effectiveness of a pre-treatment snack on uptake of mass treatment.

**Methods and Findings:**

In a cluster randomized trial carried out in Jinja district, Uganda, 12 primary schools were randomized into two groups; one received education messages for schistosomiasis prevention for two months prior to mass treatment, while the other, in addition to the education messages, received a pre-treatment snack shortly before mass treatment. Four weeks after mass treatment, uptake of praziquantel was assessed among a random sample of 595 children in the snack schools and 689 children in the non-snack schools as the primary outcome. The occurrence of side effects and the prevalence and mean intensity of *Schistosoma mansoni* infection were determined as the secondary outcomes. Uptake of praziquantel was higher in the snack schools, 93.9% (95% CI 91.7%–95.7%), compared to that in the non-snack schools, 78.7% (95% CI 75.4%–81.7%) (*p* = 0.002). The occurrence of side effects was lower in the snack schools, 34.4% (95% CI 31.5%–39.8%), compared to that in the non-snack schools, 46.9% (95% CI 42.2%–50.7%) (*p* = 0.041). Prevalence and mean intensity of *S. mansoni* infection was lower in the snack schools, 1.3% (95% CI 0.6%–2.6%) and 38.3 eggs per gram of stool (epg) (95% CI 21.8–67.2), compared to that in the non-snack schools, 14.1% (95% CI 11.6%–16.9%) (*p* = 0.001) and 78.4 epg (95% CI 60.6–101.5) (*p* = 0.001), respectively.

**Conclusions:**

Our results suggest that provision of a pre-treatment snack combined with education messages achieves a higher uptake compared to the education messages alone. The use a pre-treatment snack was associated with reduced side effects as well as decreased prevalence and intensity of *S. mansoni* infection.

**Trial registration:**

www.ClinicalTrials.gov NCT01869465

*Please see later in the article for the Editors' Summary*

## Abstract 2

In a cluster randomized trial, Simon Muhumuza and colleagues examine the effectiveness of a pre-treament snack given to school-aged children on the uptake of mass treatment for schistosomiasis in Uganda.

*Please see later in the article for the Editors' Summary*

## Editors' Summary

**Background:**

Globally, more than 240 million people are infected with schistosomes, a parasitic worm found in tropical and sub-tropical fresh water. Schistosomes reproduce in snails, which release free-swimming infectious parasites that burrow into the skin of people when they wash or swim in contaminated water. Once inside a person, the parasites turn into larvae and migrate to the liver, where they become juvenile worms. These mature into 10–20 mm long adult worms and take up residence in the veins draining the gut or bladder where they mate and release eggs, some of which pass into the feces and go back into water where they hatch and infect fresh snails. Most people have no symptoms when they are first infected with schistosomes but some develop a rash or itchy skin. Later symptoms include fever, chills, cough, and muscle aches. Without treatment, schistosomiasis can persist for years, eventually causing liver, gut, bladder, and spleen damage. In Africa alone, schistosomiasis kills about 280,000 people annually.

**Why Was This Study Done?:**

Strategies for the control of schistosomiasis include the provision of clean water and adequate sanitation, and education. However, the cornerstone of control is the reduction of disease through periodic, targeted treatment with the anti-schistosomal drug praziquantel. One group that is targeted for treatment in countries affected by schistosomiasis is school-aged children. For this approach to be successful, experts recommend regular treatment of at least 75% of school-age children at risk of infection. Unfortunately, the uptake of the intervention is often low, partly because children fear praziquantel's side effects, which include diarrhea, and vomiting. The risk of developing side effects can be reduced by eating food just before taking the drug. In this cluster randomized trial (a study that compares outcomes in groups of people randomly assigned to receive different treatments), the researchers investigate whether the provision of a pre-treatment snack improves the uptake of praziquantel among school children in Jinja district of Uganda, a country that has adopted school-based mass drug administration as part of its national schistosomiasis control program. The researchers also investigated whether this intervention reduces the occurrence of side effects attributable to praziquantel, the prevalence of schistosomiasis (the proportion of the population that is infected), and the infection intensity (indicated by the density of eggs in stool).

**What Did the Researchers Do and Find?:**

The researchers randomly assigned 12 primary schools to receive education messages for 2 months before mass treatment with praziquantel or the same education messages plus a mango juice and donut snack just before treatment. The education messages included information about the dangers of schistosome infection, the importance of preventative treatment with praziquantel, and information about taking the drug with food to avoid side effects. Four weeks after mass treatment, praziquantel uptake was assessed by self report in 595 children chosen randomly from the snack schools and 689 children from the no-snack schools. Uptake of praziquantel in the snack and no-snack schools was 93.9% and 78.7%, respectively, a significant difference in outcomes that is unlikely to be a chance event. The occurrence of self-reported side effects, the prevalence of schistosome infection, and the average intensity of infection were all significantly lower in the snack schools than in the no-snack schools.

**What Do These Findings Mean?:**

These findings suggest that the provision of a pre-treatment snack combined with education messages improved uptake of mass treatment for schistosomiasis among school children in Uganda compared to education messages alone. The intervention also reduced the occurrence of side effects, the prevalence of infection, and the infection intensity. Because uptake and the occurrence of side effects were determined by self-report, some children may have provided socially desirable answers. That is, they may have said they took the drug when they didn't because they knew that is what the researchers wanted to hear. However, the infection prevalence and intensity findings validate the self-reported uptake. The researchers conclude that the provision of a snack to mitigate the side effects of praziquantel could have motivated the children to take the treatment. If future trials show that the intervention is cost-effective, the researchers suggest that the provision of pre-treatment food should be integrated into school-based mass treatment programs for schistosomiasis control at the national level in Uganda and in similar settings elsewhere in sub-Saharan Africa.

**Additional Information:**

Please access these websites via the online version of this summary at http://dx.doi.org/10.1371/journal.pmed.1001640.

- The World Health Organization provides detailed information about schistosomiasis (in several languages)
- The US Centers for Disease Control and Prevention provides information for the public and for health professionals about all aspects of schistosomiasis (in English and Spanish)
- The UK National Health Service Choices website also provides information about schistosomiasis
- More information about the Evaluation of Strategies for Improved Uptake of Preventive Treatment for Intestinal Schistosomiasis trial is available
- The End Fund, a US not-for-profit organization that aims to tackle schistosomiasis and other neglected tropical diseases, has a personal story about dealing with schistosomiasis

## Introduction

Schistosomiasis is a major global public health problem especially in sub-Saharan Africa [1]–[3]. The disability-adjusted life-years (DALYs) loss attributable to schistosomiasis and soil transmitted helminthiasis (STHs) combined (44 million) are more than those due to malaria (36 million) and close to those due to tuberculosis (47 million) [4]–[7]. According to the Global Burden of Disease Study (2010), schistosomiasis alone is responsible for an estimated 3.3 million DALYs [8].

Control strategies recommended by WHO for long-term morbidity control of schistosomiasis include provision of safe and effective anti-schistosomal drugs such as praziquantel, and preventive measures focusing on health education, clean water, and adequate sanitation [2],[3],[9]–[11]. In many developing countries in sub-Saharan Africa including Burkina Faso, Ghana, Mali, Niger, Ivory Coast, and Tanzania, regular chemotherapy with praziquantel is implemented as the main strategy for schistosomiasis control with varying levels of success [11]–[13]. The target is to provide regular treatment of at least 75% of school-age children at risk of morbidity in order to keep the worm burden in individuals low and confined [3]. In these countries, praziquantel is mainly delivered through school-based health programs at low cost [14]–[16].

In Uganda, school-based mass drug administration (MDA) for schistosomiasis is the main approach that was adopted by the national schistosomiasis control program and is currently implemented in more than 38 districts within regions surrounding large lakes and rivers [17]. The success of the school-based strategy was based on the premise that schools are widely distributed particularly in the rural areas with high enrollment and attendance rates, that teachers are willing to administer the drugs to the children, and that the children will accept the treatment. The benefit of this approach has been documented [18]–[20]. However, studies undertaken among school children have reported low uptake of praziquantel [21]–[23]. The fear of treatment and low school-attendance rates were highlighted as some of the major contributors to the low uptake. Praziquantel is generally perceived to be a strong drug that causes transient sickness and occasional fatalities and resistance to take the drug has been reported [24]–[27]. In a particular study conducted in Jinja district in Uganda, the fear of side effects of praziquantel, lack of knowledge about schistosomiasis prevention, and lack of teacher support to distribute treatment were the major factors thought to underpin the low uptake [23].

Due to the risk of side effects and the difficulties of administering a large number of tablets to individuals, drug administration should be accompanied by precautionary measures before mass treatment is rolled out [28]. The administration of praziquantel should be accompanied by concomitant administration of food to reduce the risk of side effects of the drug [29]–[31]. Considerable reductions in occurrence of side effects and high treatment coverage have been reported by large-scale national control programs where special feeding programs for the children to mitigate the side effects were provided [31],[32]. The Ugandan Government's policy under the universal primary education program requires that parents and care takers of children take responsibility for feeding their children while at school [33],[34]. In many parts of the country, children go to school in the morning without a meal because many parents cannot afford to meet the cost of a daily meal for their children while at school [34]. This situation implies that in schools where annual mass treatment for schistosomiasis is implemented, most children take the treatment on empty stomachs with the risk of widespread side effects [23],[35],[36]. In this study, we hypothesized that provision of a pre-treatment snack in combination with education messages for schistosomiasis prevention would improve uptake as well as reduce the occurrence of side effects attributable to praziquantel. It was anticipated that the improved uptake would reduce the prevalence and intensity of schistosomiasis infection.

## Materials and Methods

### Ethical Considerations

The study protocol was approved by Makerere University College of Health Sciences Higher Degrees, Research and Ethics Committee and granted ethical clearance by the Uganda National Council for Science and Technology. One month prior to the study, the objectives and the procedures of the study were explained to the district health and education authorities and to the parents or guardians of all children in the schools through half-day meetings held at the district health headquarters and the respective schools. During the meetings held in schools, written informed consent was obtained from the parents or guardians of all the children. Assent to participate in the study was obtained from all children. Children identified with schistosomiasis and/or STH were treated with praziquantel and/or albendazole.

### Study Setting

The study was carried out in 12 primary schools in Walukuba division, Jinja district of south eastern Uganda between May and July 2013. Intestinal schistosomiasis is highly endemic in the area. School-based mass treatment for schistosomiasis is implemented in the division on an annual basis as a standalone intervention. In 2013, mass treatment was conducted in June. Prior to mass treatment, the Vector Control Division of the Ministry of Health conducted refresher training for members of the district health team and the district education office. The district then cascaded training and supervision duties to the sub-county health assistants and inspector of schools who in turn, trained and supervised the school teachers. In addition, awareness of schistosomiasis control was communicated through radio talk shows and radio spot messages. The drugs, tally sheets and registers were distributed to the primary schools after the training. During mass treatment, a central location in each school was organized for drug administration. Children were invited according to their grade, to receive treatment. Praziquantel was distributed according to the height of the child using a standard dose pole, which provides a simple and reasonably accurate estimate of weight [37]. These activities were supported through a parallel structure within the Ministry of Health with external funding from the United States Agency for International Development (USAID) channelled through Research Triangle Institute (RTI) International.

### Study Design, Stratification, and Randomization

**Figure 1:**
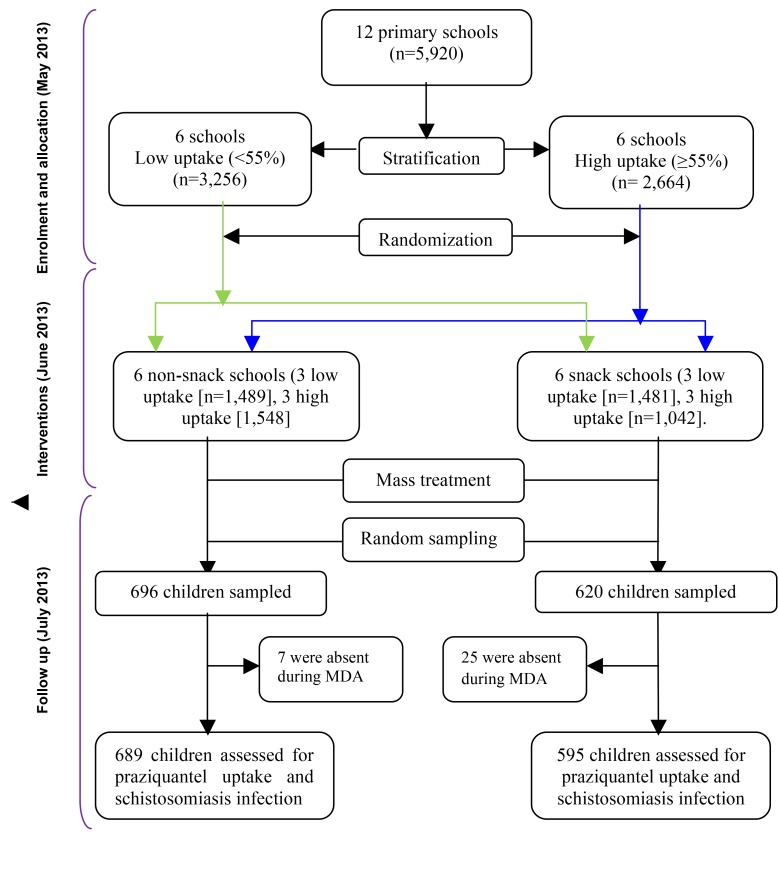
Study profile showing stratification and randomization of the schools to the intervention groups and the selection of children for assessment after mass treatment.

This was a cluster randomized trial during which 12 primary schools were grouped into two strata of six schools each according to the 2012 uptake levels of praziquantel; low (<55%) uptake and high (≥55%) uptake [38]. From each stratum, the schools (clusters) were randomized into two groups of six schools each; the snack and the non-snack group using a computer generated program in STATA 10.0 (Figure 1). A cluster design was adopted for logistical convenience since the study evaluates unblinded interventions that would otherwise cause resentment or contamination if they were to be provided for some children but not others in the same schools. Stratification was aimed at creating a good balance in participant characteristics in terms of uptake levels of praziquantel in each group (Figure 1). Randomization was performed by an independent statistician.

### The Interventions

This included a pre-treatment snack. In addition, education messages for schistosomiasis prevention were provided.

#### The pre-treatment snack

The snack consisted of mango juice and doughnuts. These were considered safe for consumption, were locally available at low cost, could be easily transported to the schools, stored, and distributed to the children during mass treatment. Each child in the snack schools received 500 ml of mango juice and a doughnut shortly before swallowing praziquantel during mass treatment. A total of 2,833 children in six primary schools received the snack. Ingredients of the mango juice included vitamin C, fruit flavors from concentrate, sugar, water, citric acid, color E 110, and preservative E 221. The 500 ml bottles of mango juice are certified by the Uganda national bureau of standards and were considered safe for human consumption. The doughnuts were made of wheat flour, baking powder, sugar, and cooking oil. A local manufacturer was contracted to make, pre-pack, and distribute the snack to the schools. The snack was distributed by the class teachers who also distributed and recorded the treatment in separate registers.

#### The education messages

In addition to the pre-treatment snack, education messages for schistosomiasis prevention were provided. These were developed by the authors in collaboration with the district health and education sectors. The messages were tailored to the knowledge gap established earlier [23] and included the following aspects: (i) dangers of schistosomiasis infection; (ii) acquisition of infection from contaminated water; (iii) prevention of acquiring the infection by avoiding unnecessary water contact; (iv) taking preventive treatment (praziquantel) annually to avoid getting serious disease; (v) taking the treatment with food in order to avoid the side effects of the drugs. The head teachers and school teachers in charge of health and sanitation were trained in the above aspects of schistosomiasis prevention through a two-day workshop, during which the print materials of the messages were also distributed. School children in both the snack and the non-snack group received a 30-minute session of the above messages, twice a week for two months, prior to mass treatment. A total of 5,920 children in the 12 primary schools received the messages. The trained teachers delivered the messages in both the local language of Lusoga and English through face to face interactions with the children during school assemblies.

#### Costs

The total cost of providing the pre-treatment snack and education messages was estimated at US$0.6 and US$0.1 per child, respectively.

### Sample Size

The sample size used in this study was based on that required to detect an increase in uptake from 49% registered in 2012 [38] to the recommended 75% [3]. At 90% power and a 95% CI, the sample size required to detect this difference was 79 in the snack schools and 79 in the non-snack schools (STATA 10.0). This sample size was adjusted by 10% non-response to 87. Because of the cluster design, a design effect of 6.3 from a previous study [23] was applied to obtain a minimum sample size of 548 in the snack schools and 548 in the non-snack schools.

### Sampling, Eligibility Criteria, and Data Collection

Sampling and data collection were conducted one month after mass drug administration. The study schools were pre-visited to obtain updated school populations. Probability proportion to size of the school and grade populations was used to determine the number of children selected from each school and grade in the two groups. Children were randomly selected from grades 4–6 using systematic sampling. Children were considered eligible for the study if they had been enrolled or attended class at the school for more than six months prior to data collection and were present at school during mass treatment. Experienced research assistants, all blinded to the children's intervention groups conducted face to face interviews with each selected child using structured questionnaires. Throughout data collection, the research assistants were not made aware of the intervention groups. Stool specimens from each child were concurrently collected, processed, and examined for *S. mansoni* infection. Children who failed to provide stool specimens for examination were replaced by randomly selecting an equal number of children from grades 4–6. A total of 12 children did not provide stool specimens and were replaced. Throughout the stool collection procedure, boys and girls were separated. The data were made available and shared with the national Vector Control Division, Ministry of Health.

### Measures

Measures were determined at both the cluster and individual levels. The primary outcome was uptake of mass treatment. The secondary outcomes were occurrence of side effects, knowledge of schistosomiasis prevention, and prevalence and intensity of *S. mansoni* infection in the two groups. In addition, the socio-demographic characteristics of the children were also assessed. The proportion of children who received education messages and a snack prior to mass treatment in the two groups was calculated. Uptake of praziquantel was defined as having received and swallowed the drug during mass treatment, which was measured through self-report. Children who reported to have swallowed treatment were asked if they developed any side effects after swallowing the drug. The socio-demographic characteristics assessed included age, sex, school name, grade (year), and distance of residence/school from the lake. Knowledge of schistosomiasis prevention was measured on a score scale of 1–3. The children were asked the different ways through which the infection can be transmitted and prevented, including taking preventive treatment. For each of the correct responses, a score of 1 was awarded. Children who mentioned at least one correct method of transmission and two correct methods of prevention scored an aggregate of 3 and were regarded as having correct knowledge of schistosomiasis prevention.

#### Stool examination for diagnosis of *S. mansoni* infection

On two consecutive days, early morning stool specimens were collected from each child. From each specimen, two slides were prepared using the modified Kato-Katz thick smear technique [39] with a 41.7 mg template. Two experienced technicians, all blinded to the children's intervention groups, examined the slides under the microscope (10×). An independent technician read a random 10% of stool slides and the results were compared. If there was a discrepancy of more than 5%, the slides were read again by two independent technicians until counts harmonized. However, all results concurred with those of the third technician. The egg counts were found to be over dispersed and thus were logarithmically transformed and intensities reported as geometric mean intensity (GMI) of eggs per gram of stool (epg) among positives only. In case of the presence of soil transmitted helminthiasis (STH) infections on the slides, this was registered so the child could be treated.

### Data Management and Statistical Analysis

Double data entry and validation was performed in EpiInfo software (version 7.1.2; Centers for Disease Control and Prevention, Atlanta (Georgia), US). After internal consistency checks, the cleaned dataset was exported to STATA 10.0 for analysis. To determine whether the groups were comparable in terms of uptake and prevalence and intensity of *S. mansoni* infection, the uptake levels and prevalence and intensity of *S. mansoni* infection in a random sample of another set of children in the same schools during a previous mass treatment of 2012 were compared. However, since the children were selected at random in both studies, it is possible that some of the children examined in 2012 could have been re-examined in 2013. Differences in the demographic and descriptive variables between the groups were compared using chi squared tests for dichotomized or categorical variables. Student's *t*-test was used to compare mean intensity of *S. mansoni* infection. To adjust for clustering, comparisons with test statistics based on chi squared tests were divided by the design effect, while test statistics based on the *t*-test were divided by the square root of the design effect [40],[41]. To evaluate the effect of the snack on uptake, a logistic regression mixed effects model allowing for fixed (school-level) and participant-level random effects was applied. ANOVA was used to compare the effect of the snack on mean intensity infection with regard to sex and age. All the analyses were planned prior to the study.

### Quality Control

Experienced research assistants who were fluent in the local language of Lusoga were recruited and trained in data collection methods. The questionnaires were translated into Lusoga and then back translated to ensure consistency in meaning. They were then pre-tested in one of the schools in a non-study area for purposes of clarity, validation, suitability, and logical flow of the questions. Interviews with the children were held in a private and quiet environment within the school premises. Data were checked for completeness and accuracy before leaving the field and consequently, no data were missing. The first author closely supervised the research assistants during data collection.

## Results

### Baseline Characteristics

A total of 1,284 children in 12 primary schools were assessed: 595 (46.3%) in the snack schools and 689 (53.7%) in the non-snack schools. The mean age was 11.3 years (standard deviation [SD] 1.7) and 11.7 years (SD 1.6) in the snack and non-snack schools, respectively. The majority 317 (53.3%) in the snack schools and 365 (53.0%) in the non-snack schools were aged between 12 and 16 years. Children in both groups were comparable in terms of age group, sex, distance from area of residence to the lake, and knowledge of schistosomiasis prevention (*p*>0.05) (Table 1). The uptake levels and prevalence and intensity of *S. mansoni* infection in a random sample of children in the same schools during a previous mass treatment of 2012 were also comparable. Uptake of praziquantel in the snack schools was 46.5% while that in the non-snack schools was 51.0% (*p* = 0.57). The prevalence of *S. mansoni* infection in snack schools was 34.4% compared to 30.9% in the non-snack schools (*p* = 0.64) and the intensity of *S. mansoni* infection was 115.9 epg and 120.5 epg in the snack and non-snack schools (*p* = 0.26), respectively (Table 2) [38].

**Table 1:** **Baseline characteristics of the two intervention groups.**

Characteristic	Non-snack Schools (*n* = 689) (%)	Snack Schools (*n* = 595) (%)	χ^2a^	*p*-Value
**Age group**				
7–11 years	291 (42.2)	310 (52.1)	1.981	0.16
12–16 years	398 (57.8)	285 (47.9)	—	—
**Sex**				
Female	343 (49.8)	296 (49.8)	0.00003	0.99
Male	346 (50.2)	299 (50.3)	—	—
**Distance from area of residence to the lake**				
≤5 km	330 (47.9)	284 (47.7)	0.0035	0.98
>5 km	359 (52.1)	311 (52.3)	—	—
**Knowledge of schistosomiasis prevention**				
Yes	595 (86.4)	500 (84.0)	0.2179	0.64
No	94 (13.6)	95 (16.0)	—	—

a Adjusted for cluster design effect.

**Table 2:** **Uptake of praziquantel, prevalence, and intensity of** ***S. mansoni*** **infection among a random sample of children in the same schools during the 2012 mass treatment.**

Characteristic	Non-snack Schools (*n* = 543) (%)	Snack Schools (*n* = 477) (%)	Test Statistic^a^	*p*-Value
**Self-reported uptake of praziquantel**				
Yes	277 (51.0)	222 (46.5)	χ^2^ = 0.322	0.57
No	266 (48.9)	255 (53.5)	—	—
***S. mansoni*** ** infection status (%)**				
Positive	168 (30.9)	164 (34.4)	χ^2^ = 0.217	0.64
Negative	375 (69.1)	313 (65.6)	—	—
**Intensity of ** ***S. mansoni*** ** infection (epg)**				
GMI epg	120.5	115.9	*t* = −1.126	0.26

a Adjusted for cluster design effect.

#### Proportion of children who received education messages and a pre-treatment snack

The majority of children in the snack 436 (73.3%) and non-snack schools 492 (71.4%) reported to have received education messages for schistosomiasis prevention prior to mass treatment (*p* = 0.766). A total of 519 (92.8%) and 270 (49.8%) children in the snack and non-snack schools, respectively, reported to have eaten something prior to mass treatment (*p*<0.001). The majority (92.7%) in the snack received mango juice and doughnuts while 48.2% in the non-snack schools reported to have taken maize porridge, 6.7% reported posho and beans, 5.2% mango juice and doughnut, and 40.0% reported other types of food including pancakes, bananas, cassava, bread, and dry tea.

### Primary Outcome

#### Self-reported uptake of praziquantel

Uptake of praziquantel in the snack and non-snack schools was 93.9% and 78.7%, respectively (*p* = 0.002).

### Secondary Outcomes

#### Occurrence of side effects and prevalence and intensity of *S. mansoni* infection

The children who reported to have developed side effects in the snack and non-snack schools were comparable in terms of age (*p* = 0.08) and sex (*p* = 0.55). The proportion of children who reported to have developed at least one side effect after swallowing praziquantel was 34.4% in the snack schools compared to 46.9% in the non-snack schools (*p* = 0.041). The occurrence of side effects among the infected children was 34 (7.6%) and that among the non-infected was 421 (92.4%) (*p* = 0.016). The most common reported side effects in both groups included abdominal pain, dizziness, diarrhea, and vomiting. Among the children who reported to have eaten something prior to mass treatment, 276 (36.3%) developed side effects compared to 163 (53.4%) among the children who did not eat anything (*p*<0.001).

The difference in prevalence of *S. mansoni* infection between the two groups was statistically significant (*p* = 0.001). The prevalence and GMI of *S. mansoni* infection was 1.3% and 38.3 epg and 14.1% and 78.4 epg in the snack and non-snack schools, respectively (Table 3). Children who reported to have taken praziquantel had a lower prevalence of infection, 60/1,101 (5.5%), compared to those who did not take praziquantel, 45/183 (24.6%) (prevalence ratio  = 1.83 [95% CI 1.60–2.10], *p*<0.001). Similarly, the GMI of infection was lower among children who reported to have taken praziquantel, 46.2 epg (95% CI 35.0–60.9) compared to those who reported not to have taken the drug, 106.1 epg (95% CI 74.7–150.6).

**Table 3:** **Characteristics of the snack and non-snack schools after the interventions.**

Variable	Non-snack Schools *n* = 689 (%)	Snack Schools *n* = 595 (%)	Test Statistic^a^	*p*-Value
**Received education messages prior to mass treatment (%)**				
Yes	492 (71.4)	436(73.3)	χ^2^ = 0.089	0.77
No	197 (28.6)	159 (26.7)	—	—
**Eaten something prior to mass treatment (%)**				
Yes	270 (49.8)	519 (92.8)	χ^2^ = 39.825	<0.001
No	272 (50.2)	40 (7.2)	—	—
**Type of food/snack taken prior to mass treatment (%)**				
Mango juice and doughnut	14 (5.2)	481 (92.7)	χ^2^ = 95.333	<0.001
Maize porridge	130 (48.2)	2 (0.4)	—	—
Posho and beans	18 (6.7)	12 (2.3)	—	—
Other (pancakes, bananas, cassava, bread, and dry tea)	108 (40.0)	24 (2.3)	—	—
**Self-reported uptake of praziquantel (%)**				
Yes	542 (78.7)	559 (93.9)	χ^2^ = 9.683	0.002
No	147 (21.3)	36 (6.1)	—	—
**Side effects attributable to praziquantel (%)**				
Yes	254 (46.9)	192 (34.4)	χ^2^ = 4.181	0.041
No	288 (53.1)	367 (63.6)	—	—
**Reported side effects attributable to praziquantel (%)**				
Abdominal pain	133 (52.4)	109 (56.8)	χ^2^ = 0.738	0.947
Dizziness	61 (24.0)	45 (23.4)	—	—
Diarrhea	31 (12.2)	27 (14.1)	—	—
Vomiting	19 (7.5)	7 (3.6)	—	—
Other (headache, nausea)	10 (3.9)	4 (2.1)	—	—
***S. mansoni*** ** infection status (%)**				
Positive	97 (14.1)	8 (1.3)	χ^2^ = 10.937	0.001
Negative	592 (85.9)	587 (98.7)	—	—
**Intensity of ** ***S. mansoni*** ** infection (epg)**				
GMI epg	78.4	38.3	*t* = 18.54	0.001

a Adjusted for cluster design effect.

### Exploratory Outcomes

Through a step-wise forward selection method, covariates with *p*<0.2 from the unadjusted model (distance from area of residence to the lake, going to the lake, having received education messages prior to mass treatment, knowledge of schistosomiasis prevention, and intervention group) were considered for inclusion in the multivariable adjusted model. The covariates retained in the final model were belonging to the snack group (adjusted odds ratio [AOR] 4.61, 95% CI 2.48–8.58, *p*<0.001) and having received education messages prior to mass treatment (AOR 13.3, 95% CI 8.29–20.80, *p*<0.001) (Table 4).

**Table 4:** **Estimated crude odds ratios and adjusted odds ratio and their 95% CI from the final logistic regression mixed-effects model.**

Variable	COR (95% CI)	*p*-Value	AOR (95% CI)	*p*-Value
**Distance from area of residence to the lake**				
≤5 km	1.59 (1.08–2.38)	0.022	1.00 (0.59–1.70)	0.99
**Going to the lake**				
Yes	1.59 (1.07–2.38)	0.022	1.52 (0.87–2.68)	0.14
**Correct knowledge of schistosomiasis prevention**				
Yes	4.44 (3.01–6.54)	<0.001	1.39 (0.68–2.94)	0.39
**Sensitized about schistosomiasis prevention**				
Yes	12.2 (7.99–18.60)	<0.001	13.1 (8.29–20.80)	<0.001
**Intervention**				
Snack group	4.19 (2.67–6.59)	<0.001	4.61 (2.48–8.58)	<0.001

Adjusted for age and sex.

COR, crude odds ratio.

## Discussion

This study found that provision of a pre-treatment snack to school children increases uptake of mass treatment for schistosomiasis. The use of a pre-treatment snack is associated with reduced side effects attributable to praziquantel. The increased uptake in the snack group is associated with reduced prevalence and intensity of schistosomiasis infection among school children.

The self-reported uptake of praziquantel by children in the snack schools (93.9%) was considerably higher than in the non-snack schools (78.7%). A comparable high coverage (94.0%) among school children was achieved by the national control program in Sierra Leone where a special feeding program for the children was provided [32]. We ascribe the observed difference in uptake between the two groups to the pre-treatment snack. The provision of food is an incentive for children to participate in school activities including health intervention programs [42],[43]. The fact that praziquantel should be taken with food to mitigate the side effects was well known by the children because it was part of the education messages given to the children prior to mass treatment. In a previous mass treatment, more than two-thirds of the children reported fear of side effects as the major reason for non uptake of praziquantel [23]. In this study, more than half of the children in the non-snack schools did not take anything prior to mass treatment while the majority (92.8%) in the snack group received a snack before swallowing the drug. Thus, provision of a snack to mitigate the side effects of praziquantel could have motivated the children to take treatment.

Praziquantel is the current drug of choice for schistosomiasis control and is virtually the only anti-schistosomal drug readily available for treatment [28],[44],[45]. The drug is given orally, is effective as a single dose for the three major species of schistosomes, has a low toxicological profile, is well tolerated, and confers some resistance to re-infection [46]. However, praziquantel causes transient side effects of the gastro-intestinal and central nervous systems especially when the drug is taken on an empty stomach. Food high in carbohydrate content increases absorption of the drug from the gastro-intestinal tract, enhances its bioavailability, and lowers the odds of side effects [29]–[31]. Praziquantel undergoes first-pass metabolism through the cytochrome P450 pathway and only small amounts reach circulation. In the presence of high carbohydrate food, hydroxylation of the drug is inhibited and the renal clearance reduced, leading to increased plasma levels [29],[30]. By disrupting calcium ions' (Ca2+) homeostasis, praziquantel increases the permeability of the schistosome membranes to Ca2+ and the resultant rapid influx of Ca2+ causes paralysis of the parasites in a contracted state. The side effects experienced after taking the drug are a result of the contents released by the dead parasites and the subsequent host immune reaction. The frequency and severity of the side effects increase with increasing parasite burden [47]. In this study, the occurrence of side effects among the infected children was lower (7.6%) than among the non-infected children (92.4%). The observed difference could be attributable to the effect of the snack. Besides, the prevalence of *S. mansoni* infection among the children who took praziquantel was generally very low (5.6%). The major side effects reported after mass treatment in this study included abdominal pain, dizziness, diarrhea, and vomiting. Similar side effects have been reported in other studies [32],[47]–[49]. The lower occurrence of the side effects in the snack schools (34.4%) compared to the non-snack schools (46.9%) is probably attributable to the pre-treatment snack. However, even in the presence of a snack, more than a third of the children in the snack group reported to have experienced the side effects. A comparable proportion of children experiencing similar side effects after taking praziquantel with a snack was reported in Ghana (28%), Tanzania (31%) [48], and in Sierra Leon (30%) [50]. The probable explanation for the occurrence of side effects even when the drug is taken with food is the presence of moderate intensity of schistosomiasis infection among the children before treatment. Because the prevalence and intensity of infection in the snack and non-snack arm were comparable during the 2012 post mass treatment, the difference in the frequency of side effects between the two groups cannot be attributed to the difference in prevalence and intensity of infection with schistosomiasis.

The efficacy of praziquantel against adult schistosomes is indisputable [20],[51]–[53], and the reduction in prevalence and intensity of schistosomiasis infection depends on achieving and maintaining high treatment coverage [3],[28]. The lower prevalence and intensity of *S. mansoni* infection in the snack schools compared to the non-snack schools is probably due to the higher uptake of praziquantel in the snack schools compared to that in the non-snack schools.

### Study Limitations

The study assessed uptake of praziquantel and side effects of the drug based on self-report. It is possible that children could have provided socially desirable answers. However, measures were undertaken to ensure fairly accurate self-report on uptake and side effects. First, the study was conducted four weeks after mass treatment, a period short enough to minimize recall bias. Secondly, the questionnaires used were pre-tested in one of the schools in a non-study area and none of the children interviewed failed to recall whether they had received treatment or not at the last mass distribution. Children were able to recall treatment because praziquantel tablets are unusually large, pungent, and unpalatable. Similar methods for measuring uptake of treatment have been successfully used in northern and south-eastern regions of Uganda [54]. Thirdly, a number of relationships corroborated evidence that self-report in our study is fairly accurate. For instance, among the children who reported uptake of praziquantel, more than 95% were able to recall and mention the true color of praziquantel and more than 96% were able to recall whether they experienced side effects with the drug or not. Lastly, the comparison of the self-reported uptake with the *S. mansoni* infection status and intensity validated the self-reported uptake as accurate. The prevalence of *S. mansoni* infections was lower in children who reported to have taken the drug compared to those who did not take the drug. Besides, the proportion of socially desirable answers was expected to be similar in both groups and should therefore have no effect on the direction of the conclusions regarding prevalence and intensity of infection attributable to the snack. Another limitation is the cost of the snack used in the study. It is not known whether alternative food such as maize porridge would be as effective as the doughnuts and mango juice and may need further testing.

### Conclusion

This study shows that provision of a pre-treatment snack can improve uptake of mass treatment as well as reduce the side effects attributable to praziquantel treatment. The increased uptake significantly reduces the prevalence and intensity of *S. mansoni* infection in this age group. A detailed cost-effectiveness analysis of provision of locally available and relatively low cost food with comparable efficacy, such as maize porridge, during mass treatment for schistosomiasis should be undertaken and if found cost-effective, provision of food should be integrated into school-based mass treatment. This strategy may be applicable at the national level and other similar settings in sub-Saharan Africa to increase treatment coverage among school children.

## Abbreviations

AOR: adjusted odds ratio
epg: eggs per gram
GMI: geometric mean intensity

## References

[1] Chitsulo L, Engels D, Montresor A, Savioli L (2000) The global status of schistosomiasis and its control. Acta Trop 77: 41–51. 10996119 10.1016/s0001-706x(00)00122-4 PMC5633072

[2] Engels D, Chitsulo L, Montresor A, Savioli L (2002) The global epidemiological situation of schistosomiasis and new approaches to control and research. Acta Trop 82: 139–146. 12020886 10.1016/s0001-706x(02)00045-1 PMC5633073

[3] WHO (2002) Prevention and control of schistosomiasis and soil-transmitted helminthiasis: report of a WHO expert committee. WHO Tech Rep Ser. Geneva: WHO. 12592987

[4] Hotez PJ, Brindley PJ, Bethony JM, King CH, Pearce EJ, et al (2008) Helminth infections: the great neglected tropical diseases. J Clin Invest 118: 1311–1321. 18382743 10.1172/JCI34261 PMC2276811

[5] Hotez PJ, Bundy DAP, Beegle K, Brooker S, Drake L, et al.. (2006) Helminth infections: soil-transmitted helminth infections and schistosomiasis. Chapter 24. Disease control priorities in developing countries. 2nd edition. Washington (D.C.): World Bank.

[6] King CH, Dickman K, Tisch DJ (2005) Reassessment of the cost of chronic helmintic infection: a meta-analysis of disability-related outcomes in endemic schistosomiasis. Lancet 365: 1561–1569. 15866310 10.1016/S0140-6736(05)66457-4

[7] Savioli L, Albonico M, Engels D, Montresor A (2004) Progress in the prevention and control of schistosomiasis and soil-transmitted helminthiasis. Parasitol Int 53: 103–113. 10.1016/j.parint.2004.01.001 15081942

[8] Murray CJ, Vos T, Lozano R, Naghavi M, Flaxman AD, et al (2012) Disability-adjusted life years (DALYs) for 291 diseases and injuries in 21 regions, 1990–2010: a systematic analysis for the Global Burden of Disease Study 2010. Lancet 380: 2197–2223. 23245608 10.1016/S0140-6736(12)61689-4

[9] El Khoby T, Galal N, Fenwick A (1998) The USAID/Government of Egypt's Schistosomiasis Research Project (SRP). Parasitol Today 14: 92–96. 17040713 10.1016/s0169-4758(97)01206-4

[10] McCullough FS, Gayral P, Duncan J, Christie JD (1980) Molluscicides in schistosomiasis control. Bull World Health Organ 58: 681–689. 6975179 PMC2395986

[11] Savioli L, Gabrielli AF, Montresor A, Chitsulo L, Engels D (2009) Schistosomiasis control in Africa: 8 years after World Health Assembly Resolution 54.19. Parasitology 136: 1677–1681. 19765347 10.1017/S0031182009991181 PMC5642868

[12] Montresor A, Cong DT, Sinuon M, Tsuyuoka R, Chanthavisouk C, et al (2008) Large-scale preventive chemotherapy for the control of helminth infection in Western Pacific countries: six years later. PLoS Negl Trop Dis 2: e278. 18846234 10.1371/journal.pntd.0000278 PMC2565698

[13] Utzinger J, Bergquist R, Shu-Hua X, Singer BH, Tanner M (2003) Sustainable schistosomiasis control—the way forward. Lancet 362: 1932–1934. 14667754 10.1016/S0140-6736(03)14968-9

[14] Brooker S, Kabatereine NB, Fleming F, Devlin N (2008) Cost and cost-effectiveness of nationwide school-based helminth control in Uganda: intra-country variation and effects of scaling-up. Health Policy Plan 23: 24–35. 18024966 10.1093/heapol/czm041 PMC2637386

[15] Nakajima H (1992) Implementing comprehensive school health education/promotion programmes. Hygie 11: 7–8. 1398679

[16] Partnership, for, Child, Development (1999) The cost of large-scale school health programmes which deliver anthelmintics to children in Ghana and Tanzania. Acta Tropica 73: 183–204. 10465058 10.1016/s0001-706x(99)00028-5

[17] Kabatereine NB, Brooker S, Tukahebwa EM, Kazibwe F, Onapa AW (2004) Epidemiology and geography of Schistosomiaisis mansoni in Uganda: implications for planning control. Trop Med Int Health 9: 372–380. 14996367 10.1046/j.1365-3156.2003.01176.x

[18] Magnussen P, Ndawi B, Sheshe AK, Byskov J, Mbwana K, et al (2001) The impact of a school health programme on the prevalence and morbidity of urinary schistosomiasis in Mwera Division, Pangani District, Tanzania. Trans R Soc Trop Med Hyg 95: 58–64. 11280068 10.1016/s0035-9203(01)90333-5

[19] Saathoff E, Olsen A, Magnussen P, Kvalsvig JD, Becker W, et al (2004) Patterns of Schistosoma haematobium infection, impact of praziquantel treatment and re-infection after treatment in a cohort of schoolchildren from rural KwaZulu-Natal/South Africa. BMC Infect Dis 4: 40. 15471549 10.1186/1471-2334-4-40 PMC524490

[20] Zhang Y, Koukounari A, Kabatereine N, Fleming F, Kazibwe F, et al (2007) Parasitological impact of 2-year preventive chemotherapy on schistosomiasis and soil-transmitted helminthiasis in Uganda. BMC Med 5: 27. 17767713 10.1186/1741-7015-5-27 PMC2014753

[21] Mafe MA, Appelt B, Adewale B, Idowu ET, Akinwale OP, et al (2005) Effectiveness of different approaches to mass delivery of praziquantel among school-aged children in rural communities in Nigeria. Acta Trop 93: 181–190. 15652332 10.1016/j.actatropica.2004.11.004

[22] Muhumuza S, Kitimbo G, Oryema-Lalobo M, Nuwaha F (2009) Association between socio economic status and schistosomiasis infection in Jinja District, Uganda. Trop Med Int Health 14: 612–619. 19392746 10.1111/j.1365-3156.2009.02273.x

[23] Muhumuza S, Olsen A, Katahoire A, Nuwaha F (2013) Uptake of preventive treatment for intestinal schistosomiasis among school children in Jinja District, Uganda: a cross sectional study. PLoS One 8: e63438. 23667617 10.1371/journal.pone.0063438 PMC3646788

[24] Allen T, Parker M (2011) The “other diseases” of the Millennium Development Goals: rhetoric and reality of free drug distribution to cure the poor's parasites. Third World Q 32: 91–117. 21591302 10.1080/01436597.2011.543816

[25] Allen T, Parker M (2012) Will increased funding for neglected tropical diseases really make poverty history? Lancet 379: : 1097–1098; author reply 1098–1100. 10.1016/S0140-6736(12)60159-7 22293367

[26] Parker M, Allen T (2011) Does mass drug administration for the integrated treatment of neglected tropical diseases really work? Assessing evidence for the control of schistosomiasis and soil-transmitted helminths in Uganda. Health Res Policy Syst 9: 3. 21211001 10.1186/1478-4505-9-3 PMC3024987

[27] Parker M, Allen T, Hastings J (2008) Resisting control of neglected tropical diseases: dilemmas in the mass treatment of schistosomiasis and soil-transmitted helminths in north-west Uganda. J Biosoc Sci 40: 161–181. 17761005 10.1017/S0021932007002301

[28] WHO (2006) Preventive chemotherapy in human helminthiasis. Coordinated use of antheliminthic drugs in control interventions: a manual for health professionals and programme managers. Geneva: World Health Organization.

[29] Castro N, Medina R, Sotelo J, Jung H (2000) Bioavailability of praziquantel increases with concomitant administration of food. Antimicrob Agents Chemother 44: 2903–2904. 10991886 10.1128/aac.44.10.2903-2904.2000 PMC90177

[30] Mandour ME, el Turabi H, Homeida MM, el Sadig T, Ali HM, et al (1990) Pharmacokinetics of praziquantel in healthy volunteers and patients with schistosomiasis. Trans R Soc Trop Med Hyg 84: 389–393. 2124391 10.1016/0035-9203(90)90333-a

[31] Njomo D, Tomono N, Muhoho N, Mitsui Y, Josyline K, et al (2010) The adverse effects of albendazole and praziquantel in mass drug administration by trained school teachers. Afr J Health Sci 17: 10–14.

[32] Hodges MH, Dada N, Warmsley A, Paye J, Bangura MM, et al (2012) Mass drug administration significantly reduces infection of Schistosoma mansoni and hookworm in school children in the national control program in Sierra Leone. BMC Infect Dis 12: 16. 22264258 10.1186/1471-2334-12-16 PMC3282666

[33] G.O.U (2008) The Education (pre-primary, primary and post-primary) Act, 2008, (Act 13 supplement). Government of Uganda. Available: planipolis.iiep.unesco.org/upload/Uganda/Uganda_EducationAct.pdf.

[34] GCNF (2006) School feeding in Uganda, 2006. Report for the Global Child Nutrition Forum. Available: http://www.gcnf.org/library/country-reports/uganda/2006- Uganda-School-Feeding.pdf. Accessed 25 August 2011.

[35] Kabatereine NB, Kemijumbi J, Ouma JH, Sturrock RF, Butterworth AE, et al (2003) Efficacy and side effects of praziquantel treatment in a highly endemic Schistosoma mansoni focus at Lake Albert, Uganda. Trans R Soc Trop Med Hyg 97: 599–603. 15307437 10.1016/s0035-9203(03)80044-5

[36] Ndyomugyenyi R, Kabatereine N (2003) Integrated community-directed treatment for the control of onchocerciasis, schistosomiasis and intestinal helminths infections in Uganda: advantages and disadvantages. Trop Med Int Health 8: 997–1004. 14629766 10.1046/j.1360-2276.2003.01124.x

[37] Hall A, Nokes C, Wen ST, Adjei S, Kihamia C, et al (1999) Alternatives to bodyweight for estimating the dose of praziquantel needed to treat schistosomiasis. Trans R Soc Trop Med Hyg 93: 653–658. 10717759 10.1016/s0035-9203(99)90087-1

[38] Muhumuza S, Katahoire A, Nuwaha F, Olsen A (2013) Increasing teacher motivation and supervision is an important but not sufficient strategy for improving praziquantel uptake in Schistosoma mansoni control programs: serial cross sectional surveys in Uganda. BMC Infect Dis 13: 590. 24330594 10.1186/1471-2334-13-590 PMC3866576

[39] Katz N, Chaves A, Pellegrino J (1972) A simple device for quantitative stool thick-smear technique in Schistosomiasis mansoni. Rev Inst Med Trop Sao Paulo 14: 397–400. 4675644

[40] Campbell M, Grimshaw J, Steen N (2000) Sample size calculations for cluster randomised trials. Changing Professional Practice in Europe Group (EU BIOMED II Concerted Action). J Health Serv Res Policy 5: 12–16. 10787581 10.1177/135581960000500105

[41] Donner A (1998) Some aspects of the design and analysis of cluster randomized trials. Appl Statist 47: 95–113.

[42] Acham H, Kikafunda JK, Malde MK, Oldewage-Theron WH, Egal AA (2012) Breakfast, midday meals and academic achievement in rural primary schools in Uganda: implications for education and school health policy. Food Nutr Res 56.. 10.3402/fnr.v56i0.11217 PMC3280795 22347147

[43] Pollitt E, Gersovitz M, Gargiulo M (1978) Educational benefits of the United States school feeding program: a critical review of the literature. Am J Public Health 68: 477–481. 347957 10.2105/ajph.68.5.477 PMC1653885

[44] Beck L, Favre TC, Pieri OS, Zani LC, Domas GG, et al (2001) Replacing oxamniquine by praziquantel against Schistosoma mansoni infection in a rural community from the sugar-cane zone of Northeast Brazil: an epidemiological follow-up. Mem Inst Oswaldo Cruz 96 Suppl: 165–167 10.1590/s0074-02762001000900025 11586444

[45] Botros S, El-Lakkany N, Seif el-Din SH, Sabra AN, Ibrahim M (2011) Comparative efficacy and bioavailability of different praziquantel brands. Exp Parasitol 127: 515–521. 21044626 10.1016/j.exppara.2010.10.019

[46] Frohberg H, Schulze Schencking M (1981) Toxicological profile of praziquantel, a new drug against cestode and schistosome infections, as compared to some other schistosomicides. Arzneimittelforschung 31: 555–565. 7195246

[47] Stelma FF, Talla I, Sow S, Kongs A, Niang M, et al (1995) Efficacy and side effects of praziquantel in an epidemic focus of Schistosoma mansoni. Am J Trop Med Hyg 53: 167–170. 7677219 10.4269/ajtmh.1995.53.167

[48] Brooker S, Marriot H, Hall A, Adjei S, Allan E, et al (2001) Community perception of school-based delivery of anthelmintics in Ghana and Tanzania. Trop Med Int Health 6: 1075–1083. 11737845 10.1046/j.1365-3156.2001.00806.x

[49] N'Goran EK, Gnaka HN, Tanner M, Utzinger J (2003) Efficacy and side-effects of two praziquantel treatments against Schistosoma haematobium infection, among schoolchildren from Cote d'Ivoire. Ann Trop Med Parasitol 97: 37–51. 12662421 10.1179/000349803125002553

[50] Hodges MH, Sonnie M, Turay H, Conteh A, Maccarthy F, et al (2012) Maintaining effective mass drug administration for lymphatic filariasis through in-process monitoring in Sierra Leone. Parasit Vectors 5: 232. 23062561 10.1186/1756-3305-5-232 PMC3503583

[51] De Clercq D, Vercruysse J, Kongs A, Verle P, Dompnier JP, et al (2002) Efficacy of artesunate and praziquantel in Schistosoma haematobium infected schoolchildren. Acta Trop 82: 61–66. 11904104 10.1016/s0001-706x(02)00003-7

[52] Doenhoff M, Kimani G, Cioli D (2000) Praziquantel and the control of schistosomiasis. Parasitol Today 16: 364–366. 10951592 10.1016/s0169-4758(00)01749-x

[53] Frenzel K, Grigull L, Odongo-Aginya E, Ndugwa CM, Loroni-Lakwo T, et al (1999) Evidence for a long-term effect of a single dose of praziquantel on Schistosoma mansoni-induced hepatosplenic lesions in northern Uganda. Am J Trop Med Hyg 60: 927–931. 10403322 10.4269/ajtmh.1999.60.927

[54] Parker M, Allen T (2012) Will Mass Drug Administration Eliminate Lymphatic Filariasis? Evidence from Northern Coastal Tanzania. J Biosoc Sci: 1–29. 10.1017/S0021932012000466 PMC3666211 23014581

